# Impact of High Solar UV Radiant Exposures in Spring 2020 on SARS‐CoV‐2 Viral Inactivation in the UK^†^

**DOI:** 10.1111/php.13401

**Published:** 2021-03-05

**Authors:** Rebecca Rendell, Marina Khazova, Michael Higlett, John O’Hagan

**Affiliations:** ^1^ Centre for Radiation, Chemicals and Environmental Hazards Public Health England Chilton, Didcot Oxfordshire UK

## Abstract

Potential for SARS‐CoV‐2 viral inactivation by solar UV radiation in outdoor spaces in the UK has been assessed. Average erythema effective and UV‐A daily radiant exposures per month were higher (statistically significant, *P* < 0.05) in spring 2020 in comparison with spring 2015–2019 across most of the UK, while irradiance generally appeared to be in the normal expected range of 2015–2019. It was found that these higher radiant exposures may have increased the potential for SARS‐CoV‐2 viral inactivation outdoors in April and May 2020. Assessment of the 6‐year period 2015–2020 in the UK found that for 50–60% of the year, that is most of October to March, solar UV is unlikely to have a significant (at least 90% inactivation) impact on viral inactivation outdoors. Minimum times to reach 90% and 99% inactivation in the UK are of the order of tens of minutes and of the order of hours, respectively. However, these times are best case scenarios and should be treated with caution.

## Graphical abstract

Assessment of the 6‐year period 2015–2020 has shown that for around half the year in the UK solar UV is unlikely to have a significant impact on the inactivation of SARS‐CoV‐2 outdoors, with 90% inactivation not being possible even with a full day of sun exposure. The shortest times to reach 90% and 99% SARS‐CoV‐2 inactivation in the UK are of the order of tens of minutes and of the order of hours, respectively.

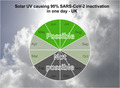

## INTRODUCTION

Spring 2020 was exceptional in the UK. In the context of the coronavirus disease (COVID‐19) pandemic, new severe acute respiratory syndrome coronavirus 2 (SARS‐CoV‐2) infections in the UK increased rapidly from early March reaching a peak in April and then slowly decreased in May (1). At the same time, spring 2020 was the sunniest on record (2), which may have reduced outdoor viral load since solar ultraviolet (UV) radiation, in particular the shortest wavelengths, is the primary virucidal agent in the environment (3, 4, 5, 6). However, sunshine hours are defined by all incident terrestrial solar radiation wavelengths (7) of which only a small proportion is in the UV wavelength range, so increases in sunshine hours do not provide quantitative information on the increases in the UV region.

Analysis using satellite data (6) shows that solar UV has potential to inactivate viruses from the coronaviridae family and that the level of inactivation varies widely depending on location and season. However, detailed analysis using ground‐based data relevant for the UK has not yet been published.

Public Health England (PHE) has a network of ground‐based solar monitoring sites across the UK and overseas and additional spectral solar monitoring capabilities at Chilton, UK. These are used for health research and the development of advice regarding sun exposure (8, 9, 10, 11, 12).

In this paper, ground‐based UK solar UV data are analyzed to determine whether significant increases in solar UV were observed in spring 2020 and if so whether this would be likely to have increased viral inactivation outdoors in comparison with spring 2015–2019. Data from the full six‐year period 2015–2020 is then analyzed in order to determine the periods when solar UV is likely to contribute to viral inactivation. In addition, the diurnal variation in viral inactivation is considered.

## MATERIALS AND METHODS

Spring 2020 in the UK was the sunniest on record, with 626 sunshine hours, 71 h greater than the previous record in 1948 (2) and 30% more than the average of the preceding 5 years (13) (see Table 1).

**Table 1:** UK sunshine hours in 2020, (13).

	Sunshine hours		
	2020	2015–2019 average	% difference
March	136.3	111.1	23%
April	224.5	166.1	35%
May	265.5	203.5	30%
Total (spring)	626.3	480.7	30%

Sunshine hours are defined by incident direct solar radiation of > 120 W m^−2^ (7). Since only a small proportion of terrestrial solar radiation is in the UV wavelength range (280–400 nm), these increases in sunshine hours cannot provide quantitative information on the variation in the UV region.

**Figure 1:**
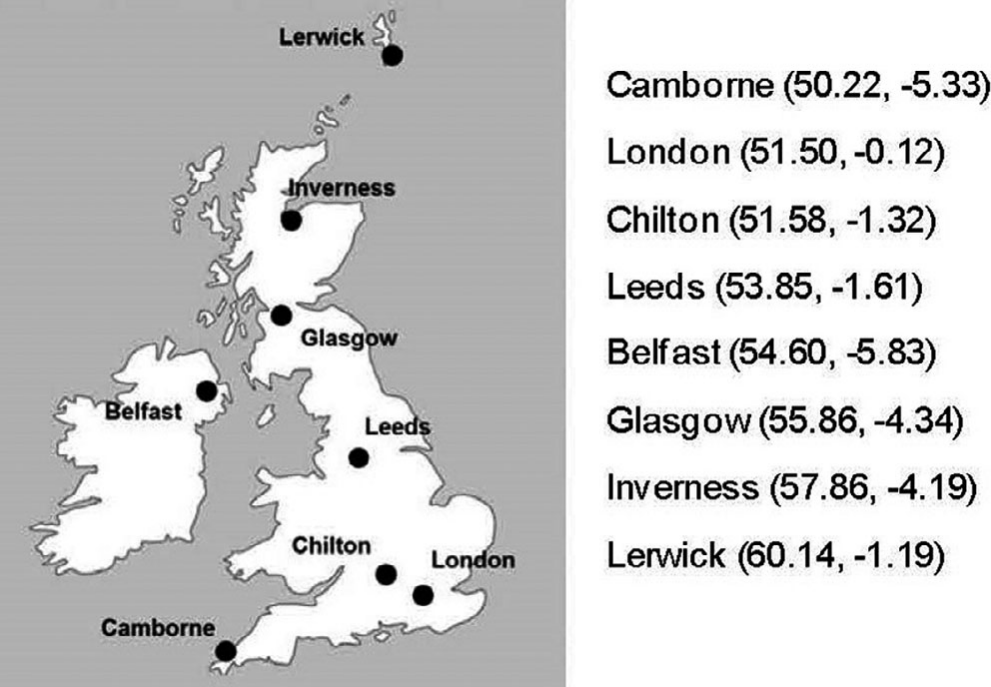
Eight of the PHE solar network sites with latitude and longitude.

The PHE solar network sites (14, 15, 16) measure UV‐A (315–400 nm) and erythema effective irradiances (17) and record these data at five minute intervals. This study utilizes data from eight of the solar network sites in the UK, with latitudes ranging from 50.22 °N to 60.14 °N, see Fig. 1.

In order to determine whether there were significant increases in solar UV in spring 2020 in the UK, erythema effective and UV‐A daily radiant exposures (doses) were calculated for March–May 2015–2020 for each of the PHE solar monitoring stations. The average daily radiant exposure for each month in spring 2020 was compared to the 2015–2019 average daily radiant exposure for the corresponding month. Statistical significance was calculated using the t‐test with a significance level of *P* < 0.05. In addition, maximum erythema effective and UV‐A irradiances for March–May 2020 were compared to the range of monthly maxima values for 2015–2019.

In order to determine whether increases in solar UV levels in spring 2020 may have increased viral inactivation outdoors in comparison with spring 2015–2019, and to determine periods of the year when solar UV is likely to contribute to viral inactivation, the erythema effective irradiance from the PHE solar network needs to be converted to viral inactivation weighted data.

The viral inactivation action spectrum was determined from (3).

Spectral irradiance measured with a Bentham DTMc300 double grating monochromator (Bentham Instruments, Reading, UK) over a wide range of solar elevation angles was used to calculate a conversion from erythema effective irradiance to viral inactivation weighted values by applying erythema (17) and viral inactivation (3) weighting functions to spectral irradiance data. This allowed erythema effective irradiance from the PHE solar network to be converted to viral inactivation weighted irradiance. From these data, times to reach thresholds of inactivation could be determined. The standard inactivation threshold used is D_90_, the fluence threshold for 90% viral inactivation. The D_90_ threshold used in this study is 6.9 J m^−2^ from (18) for 254 nm equivalent UV required for 90% inactivation of SARS‐CoV‐2. This fluence (spherical surface) threshold has been assumed to be applicable to radiant exposure (flat surface) since particles with potential for person to person transmission will normally be on surfaces or airborne but near the ground with low albedo.

Since 90% viral inactivation may not be sufficient to reduce the risk of infection, 99% inactivation has also been considered. In this study, a D_99_ threshold of 27.6 J m^−2^ has been used—four times the fluence, as given in (18). The time to reach 6.9 and 27.6  Jm^−2^ viral inactivation weighted radiant exposures, that is D_90_ and D_99_, has been calculated for each month of the year for 2015–2020 under all weather conditions. Time of day is in UTC.

The proportion of days when D_90_ and D_99_ could be reached in a single whole day and 1 h has been calculated for all sites for 2015–2020. Any years or months with more than 10% or 20% incomplete data respectively have been removed from the analysis. In addition, further detailed analysis on the diurnal variation in the time to reach D_90_ and D_99_ has been carried out on five days at Chilton.

## RESULTS AND DISCUSSION

### Spring 2020 solar UV versus spring 2015‐2019

The majority of the solar monitoring sites had statistically significant increases in average erythema effective and UV‐A daily radiant exposures in March–May 2020 compared with March–May 2015–2019 as shown in Tables 2 and 3 for mean values, range and the percentage difference between 2020 and 2015–2019 monthly average daily radiant exposures. Erythema effective radiant exposures are expressed in SEDs (100 J m^−2^) (17).

1. In March, 4 of 8 sites had statistically significant increases in erythema effective daily radiant exposure of 20% or more compared to 2015–2019 average and 4 of 8 sites had statistically significant increases in UV‐A daily radiant exposure with three of these being greater than 20%.
2. In April, all sites had statistically significant increases in erythema effective daily radiant exposure of at least 30% compared to 2015–2019 average and 7 of 9 sites had statistically significant increases in UV‐A daily radiant exposure with five of these being 20% or more.
3. In May, all sites except Lerwick had statistically significant increases in erythema effective daily radiant exposure of 20% or more compared to 2015–2019 average and five sites had statistically significant increases in UV‐A daily radiant exposure with four of these being 20% or more.
4. The sites with the greatest increases in erythema effective daily radiant exposure were Belfast (+57% in April), Camborne (+56% in May) and Inverness (+54% in April). The sites with the greatest increases in UV‐A daily radiant exposure were Belfast (+35% in April), London and Camborne (both + 32% in May).

**Table 2:** Erythema effective daily radiant exposures for 2020 and 2015‐2019 (SED).

	March			April			May		
	2015–2019 mean (range)	2020	% change	2015–2019 mean (range)	2020	% change	2015–2019 mean (range)	2020	% change
Camborne	6.9 (0.4–19.4)	9.5 (2.8–18.1)	38%*	15.1 (4.0–28.0)	20.1 (10.6–29.0)	33%*	21.3 (3.0–44.5)	33.2 (11.4–45.1)	56%*
London	6.8 (0.7–14.6)	8.2 (0.9–14.7)	20%	13.9 (2.0–25.2)	18.1 (4.5–25.1)	30%*	21.2 (4.8–37.1)	28.8 (13.6–42.0)	36%*
Chilton	7.9 (1.5–17.1)	9.7 (1.3–18.0)	23%*	15.8 (2.9–30.7)	20.9 (5.0–29.9)	32%*	22.8 (4.2–42.7)	33.2 (9.7–43.9)	46%*
Leeds	6.0 (1.1–15.2)	7.7 (2.4–14.4)	27%*	12.6 (2.2–25.8)	18.7 (5.9–28.7)	48%*	19.3 (3.9–37.3)	27.6 (10.7–43.7)	43%*
Belfast	5.5 (0.8–13.6)	7.0 (1.8–13.4)	27%*	11.5 (2.1–23.3)	18.0 (7.3–25.9)	57%*	19.8 (5.7–39.7)	24.9 (11.3–41.8)	25%*
Glasgow	5.0 (1.1–12.0)	5.6 (2.5–11.3)	11%	11.0 (2.1–22.6)	16.4 (5.8–24.0)	49%*	18.9 (3.7–36.1)	23.3 (5.9–39.2)	23%*
Inverness	5.1 (0.7–11.8)	5.6 (2.9–11.4)	10%	11.6 (2.0–23.8)	17.8 (7.7–23.5)	54%*	18.0 (2.5–36.3)	23.1 (9.6–39.2)	29%*
Lerwick	3.8 (0.7–9.0)	4.4 (0.9–8.9)	15%	9.9 (1.8–18.6)	13.0 (4.2–22.4)	31%*	16.6 (3.1–32.3)	19.4 (8.9–36.9)	17%

* Statistically significant (t‐test, *P* < 0.05).

**Table 3:** UV‐A daily radiant exposures for 2020 and 2015–2019 (kJm^−2^)

	March			April			May		
	2015–2019	2020	% change	2015–2019 mean (range)	2020	% change	2015–2019	2020	% change
Camborne	511.3 (69.4–988.5)	599.7 (144.8–969.4)	+17%	855.4 (216.5–1263.6)	934.9 (512.6–1237.6)	+9%	1007.1 (263.4–1604.2)	1326.1 (547.7–1668.0)	+32%*
London	455.8 (81.7–881.1)	570.7 (83.6–868.5)	+25%*	744.2 (147.2–1220.0)	931.8 (251.0–1274.4)	+25%*	981.8 (238.3–1534.3)	1292.7 (653.0–1635.5)	+32%*
Chilton	499.4 (123.1–935.3)	585.1 (101.1–872.0)	+17%*	792.7 (178.7–1287.6)	913.4 (251.7–1277.1)	+15%*	993.4 (233.8–1553.5)	1279.4 (468.5–1544.8)	+29%*
Leeds	438.9 (89.5–874.0)	525.0 (284.1–837.2)	+20%*	725.7 (139.7–1151.4)	879.5 (316.9–1267.5)	+21%*	935.1 (189.1–1518.1)	1127.1 (523.4–1552.9)	+21%*
Belfast	412.6 (93.4–808.2)	508.8 (166.4–819.6)	+23%*	641.4 (168.1–1172.5)	863.1 (382.1–1170.7)	+35%*	871.8 (278.8–1451.2)	1035.8 (476.7–1560.8)	+19%*
Glasgow	396.9 (100.8–735.0)	416.2 (186.7–723.0)	+5%	665.1 (150.0–1299.3)	795.4 (302.1–1140.7)	+20%*	923.3 (249.6–1499.3)	992.7 (262.0–1498.8)	+8%
Inverness	425.7 (76.9–780.0)	450.4 (285.2–755.8)	+6%	700.8 (149.7–1222.7)	898.4 (456.3–1229.9)	+28%*	903.8 (190.5–1445.4)	1006.7 (456.8–1548.1)	+11%
Lerwick	367.5 (80.2–787.9)	383.4 (104.3–653.1)	+4%	670.8 (150.6–1152.9)	671.7 (217.7–1087.7)	+0%	892.1 (185.4–1455.2)	912.4 (412.3–1510.6)	+2%

* Statistically significant (t‐test, *P* < 0.05).

It is notable that the biggest increases were seen at the shortest UV wavelengths, as indicated by the large increases in erythema effective values and that these are greater than the increases seen in UV‐A. The shortest UV wavelengths are also significantly more effective in inactivation of viruses (3).

While UV daily radiant exposures, particularly in April and May 2020, were significantly higher than the 2015–2019 average, monthly maximum erythema effective and UV‐A irradiances in spring 2020 were generally in the normal expected range of 2015–2019. For example, the three sites and months with the greatest increase in erythema effective UV daily radiant exposure had either small or negative changes in maximum erythema effective irradiance per month of +3.2%, −9.6% and −4.9% for Belfast (April), Camborne (May) and Inverness (April), respectively. Similarly, the three sites and months with the greatest increase in UV‐A daily radiant exposure had small or negative changes in maximum UV‐A irradiance per month of +1.6%, −5.9% and −3.3% for Belfast (April), Camborne (May) and London (May), respectively. One exception is Lerwick in March which, although its change in maximum UV‐A irradiance is negligible (−1.1%), saw a change in maximum erythema effective irradiance of +45.8% in March 2020. This occurred on the last day of March which is a month where erythema effective irradiance increases rapidly. Specific cloud conditions causing a brief spike in erythema effective irradiance (14) could have contributed to this outlier. The full range of changes to maximum erythema effective irradiance is from −13.5% (London, March) to +18% (Leeds, May) and for UV‐A the full range is from −11.9% (Glasgow, May) to +8.2% (Leeds, May).

It is evident that there were significant increases in solar UV in spring 2020 in comparison with spring 2015–2019—however, these were increases in radiant exposure and not irradiance. There was a higher frequency of periods of high irradiance (i.e. more periods of time with clear sunny weather) rather than exceptional increases in peak irradiance.

### Spring 2020—viral inactivation

**Figure 2:**
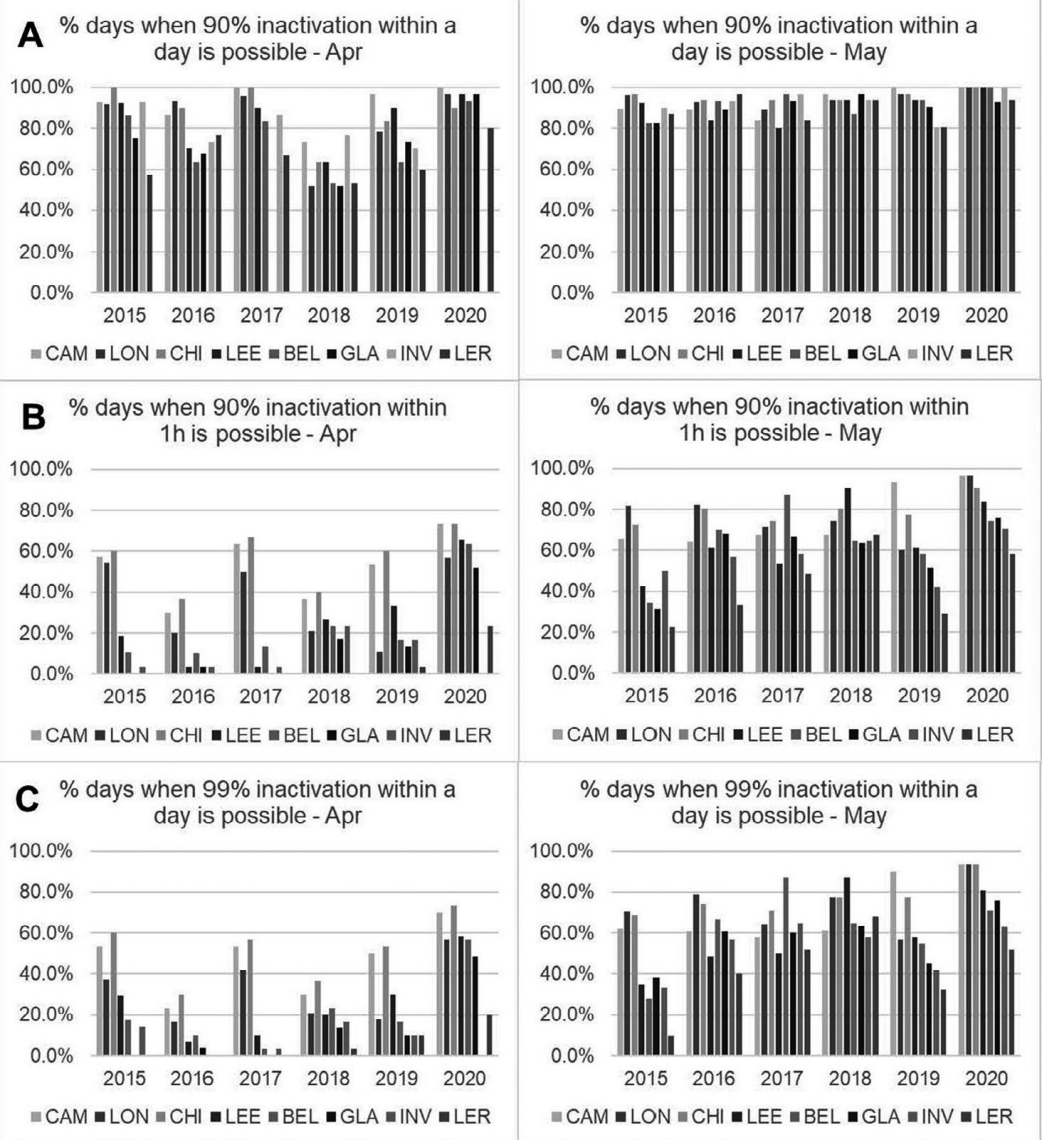
Percentage of days in April and May 2015–2020 when D_90_ and D_99_ could be reached within a day and when D_90_ could be reached within 1 h. D_99_ could not be reached within 1 h for any day or any UK location. For March, D_90_ could not be reached within a day on most days (see Table 4) and the number of days when D_90_ could be reached in 1 h and D_99_ could be reached in 1 day was negligible (mean and range 0.1% (0.0–2.2%) and 0.1% (0.0–1.4%), respectively).

For a threshold of reaching D_90_ within a day, the significant increases in solar UV radiant exposure in spring 2020 in comparison with spring 2015–2019 appear to have the greatest effect in April (Fig. 2A, Table 4). In March, the proportion of days on which D_90_ could be reached within a day was generally < 5% for 2015‐2019 and was still < 5% for all sites in 2020. In contrast, by May D_90_ could be reached in a single day on the majority of days in 2015–2019 and in 2020. For D_90_ being reached within 1 h and the higher threshold D_99_ being reached within a day, the significant increases in solar UV radiation exposure in spring 2020 in comparison with spring 2015–2019 appear to have an impact in both April and May, with a negligible proportion of days reaching these thresholds in March in any year. This ties in with the previous finding that there were significant increases in solar UV in spring 2020 in comparison with 2015–2019 but the increases were in radiant exposure and not peak irradiance.

**Table 4:** Percentage of days when D_90_ could be reached in 1 day for March, April and May 2020 *versus* 2015–2019.

Latitude	Site	Full year		Mar		Apr		May	
		2015–2019 range (%)	2020 (%)	2015–2019 range (%)	2020 (%)	2015–2019 range (%)	2020 (%)	2015–2019 range (%)	2020 (%)
50.22	CAM	48.2–54.9	55.9	1.4–5.2	3.6	73.3–100.0	100.0	83.9–100.0	100.0
51.50	LON	46.3–51.5	54.4	0.5–3.3	3.8	51.7–95.8	96.7	89.3–96.7	100.0
51.58	CHI	50.8–55.6	54.5	1.4–4.7	4.6	63.3–100.0	90.0	93.5–96.8	100.0
53.85	LEE	45.4–48.8	53.8	1.1–3.3	3.3	63.3–92.6	96.6	80.0–93.5	100.0
54.60	BEL	42.7–48.1	51.2	0.0–12.9	2.5	53.3–86.2	93.3	82.8–96.8	100.0
55.86	GLA	40.6–46.1	49.0	0.5–1.1	1.7	51.7–75.0	96.6	82.8–96.7	93.1
57.86	INV	42.5–46.7		0.0–2.2	0.8	70.0–92.9		80.6–96.8	100.0
60.14	LER	36.2–39.9	44.0	0.0–0.8	1.1	53.3–76.7	80.0	80.6–96.7	93.5

### Viral inactivation during the 6‐year period 2015‐2020

**Figure 3:**
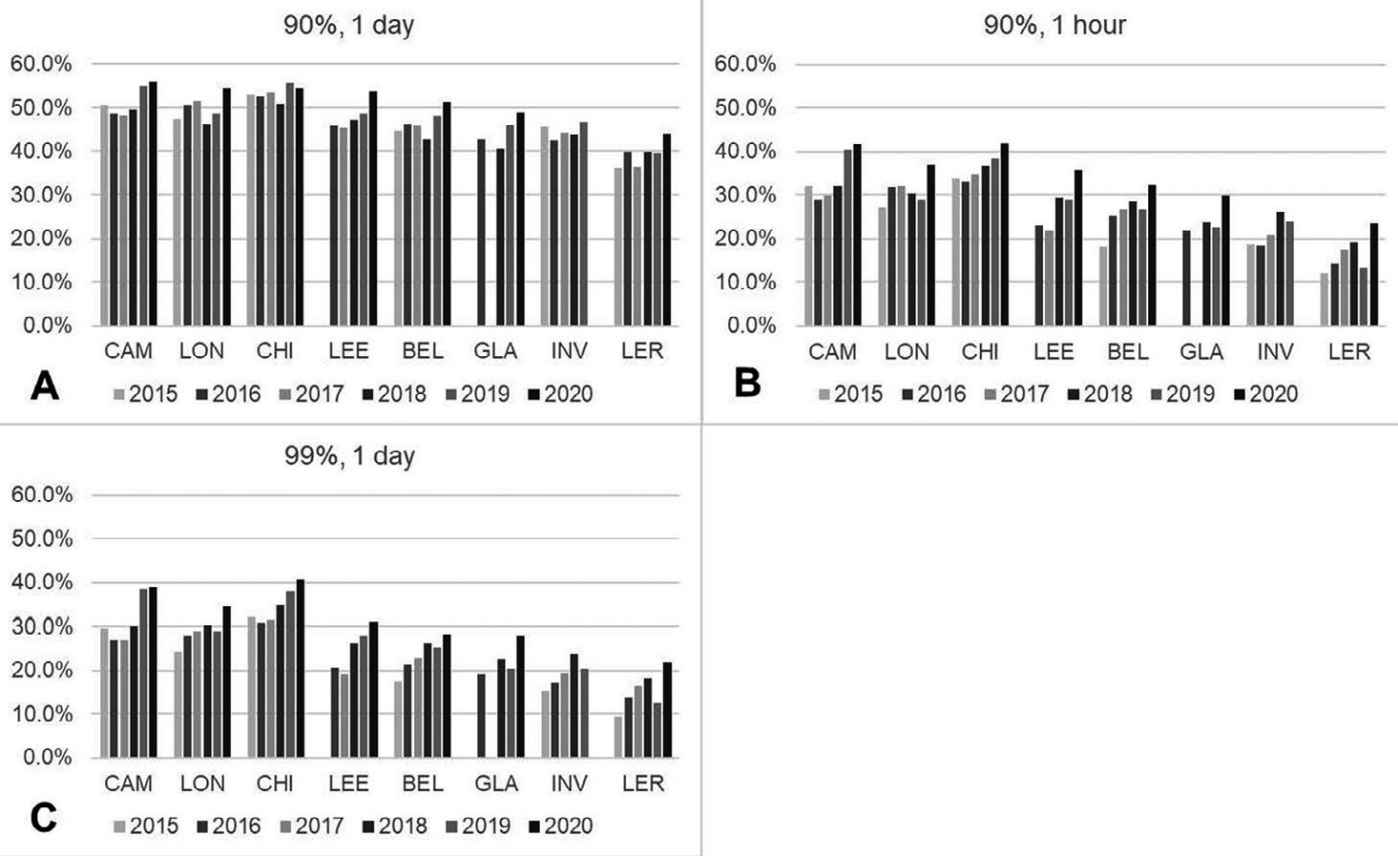
Percentage of days per year, 2015–2020, when D_90_ and D_99_ could be reached within a day and when D_90_ could be reached within an hour. D_99_ could not be reached within an hour for any day.

Figure 3A shows that across a whole year, D_90_ could be reached within a day on around 40% of days at the highest UK latitudes and around 50% of days at the lowest UK latitudes. There was larger variation across the latitudes for D_90_ being reached within an hour, ranging from 10% to 20% of days per year at the highest latitudes to 30–40% of days at the lowest latitudes (Fig. 3B). This distribution is very similar for the percentage of days when D_99_ could be reached within a day (Fig. 3C). It was found that D_99_ could not be reached within an hour on any day for any site or year in the UK. These findings also show that a viral inactivation threshold of D_90_ or greater could not be reached even with a full day of exposure to solar UV for 50‐60% of the year (lowest–highest UK latitudes).

**Figure 4:**
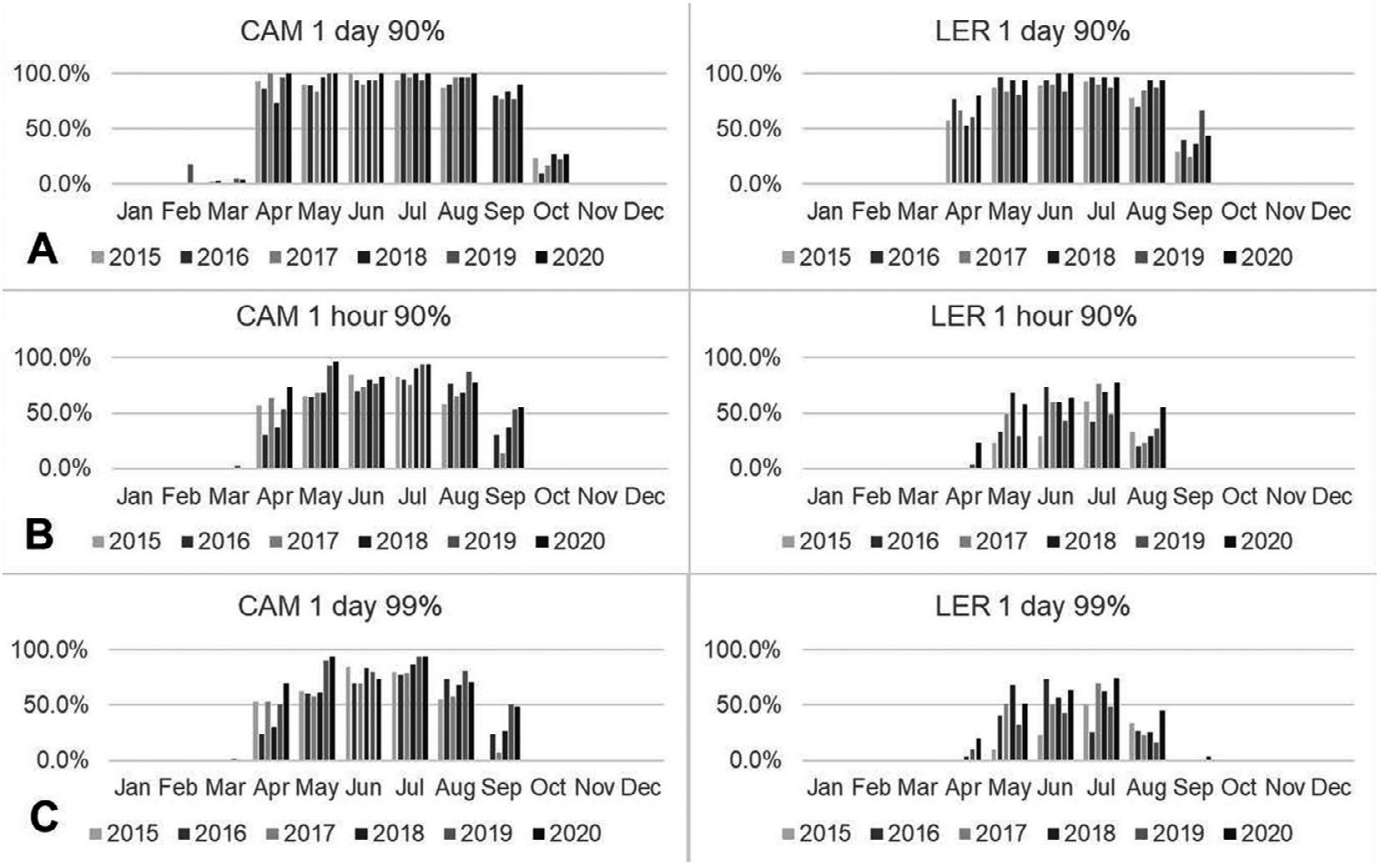
Proportion of days when D_90_ and D_99_ could be reached within a single day and when D_90_ could be reached within 1 h at the lowest (Camborne, 50.22 °N) and highest (Lerwick, 60.14 °N) latitude sites in the UK across the year.

Across the months of the year, it was found that D_90_ could be reached within 1 day from April to September across the UK, with approaching 100% of days reaching this threshold in May, June and July. Conversely, for most of October–March, and all of November–January, D_90_ could not be reached within a day (Fig. 4A). For reaching D_90_ within 1 h or the higher threshold of reaching D_99_ within a single day, which show a similar pattern to each other, these thresholds could be reached from April to September at the lower latitudes of the UK for up to around 75% of days. At higher latitudes, these could generally only be reached from May to August for up to around 50% of days (Fig. 4B and C).

### Diurnal variation in time to reach D_90_ and D_99_

**Figure 5:**
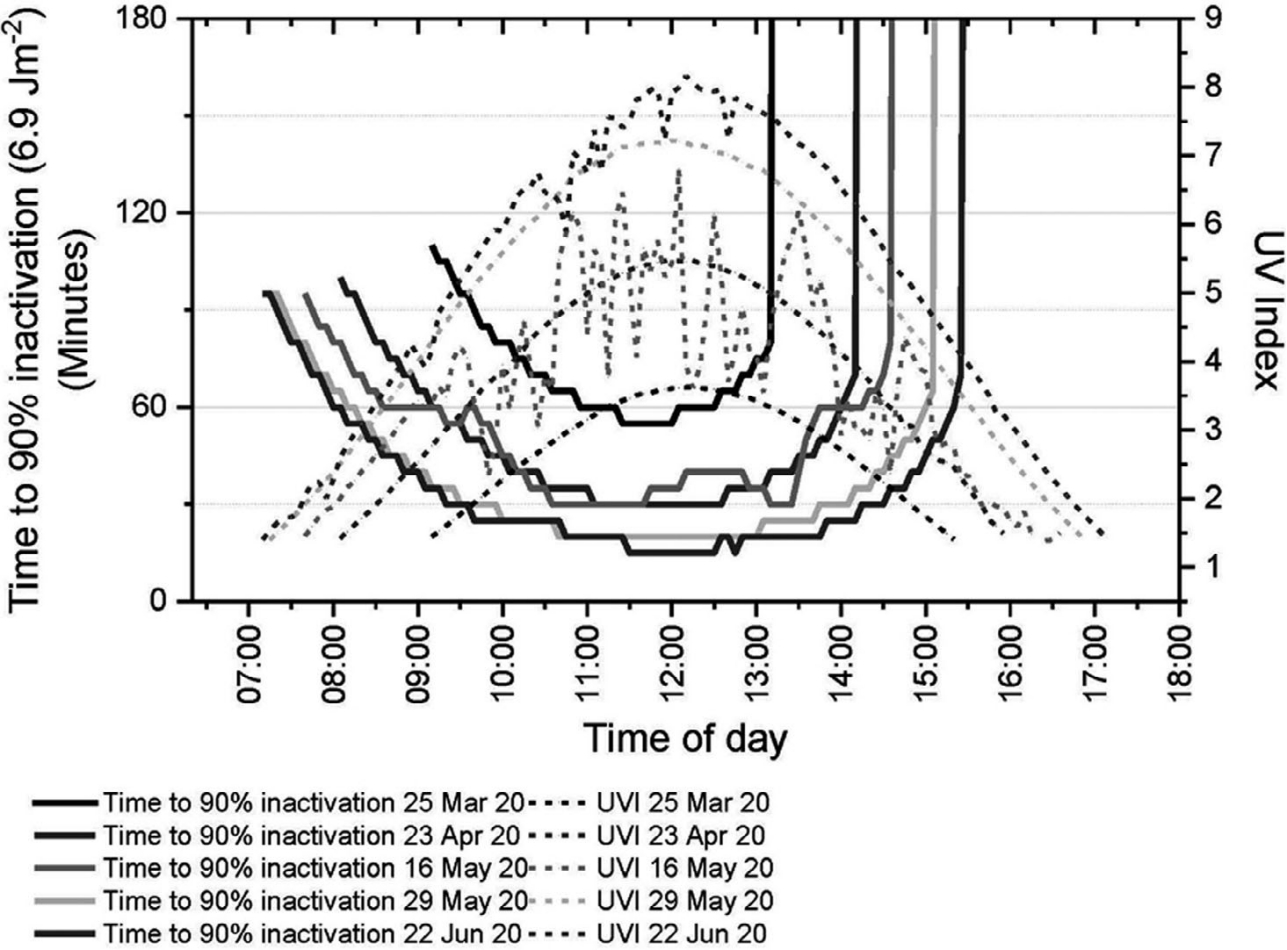
UV Index and time to reach D_90_ (best case scenarios) for three clear days (25 March, 23 April, 29 May), one cloudy day (16 May) in spring and one relatively clear day near the summer solstice (22 June) in Chilton in the south of the UK. Time of day is in UTC. The time of day at which the inactivation times run vertically off the graph in the afternoon is the time from which reaching D_90_ would not be possible on the same day.

Figure 5 shows the diurnal variation in the time to reach D_90_ at Chilton in the south of the UK (see Fig. 1) on one clear day in March, April and May 2020, on one variably cloudy day in May 2020 and also on the relatively clear day of 22 June 2020 (very near the summer solstice) which reaches the highest UV Index generally expected to be seen in the UK.

Figure 5 shows that the time to reach D_90_ is highly dependent on time of day and time of year, with the shorter times to reach D_90_ being around or just before noon and on days nearer the summer solstice. Figure 5 also shows that clear, sunny days have greater effectiveness at viral inactivation than cloudy days. It can be seen that the time to reach D_90_ on the variably cloudy day with sunny spells of 16 May 2020 is comparable to reach D_90_ on the clear day of 23 April 2020, 23 days earlier.

The shortest time to reach D_90_ and D_99_ for each of these days and the cutoff time—the time from which the threshold cannot be reached—are shown in Table 5.

**Table 5:** Shortest times required to reach D_90_ and D_99_ SARS‐CoV‐2 inactivation at Chilton in the south of the UK. Time of day is in UTC.

	D_90_			D_99_		
	Shortest time to reach threshold (minutes)	Starting from	Cutoff (threshold cannot be reached from this time)	Shortest time to reach threshold	Starting from	Cutoff (threshold cannot be reached from this time)
25 Mar 20	55	11:25–12:05	13:15	4 h 55	09:40–09:50	10:15
23 Apr 20	30	11:05–12:35	14:15	2 h 15	10:30–11:25	12:25
16 May 20	30	10:35–11:45, 13:05–13:25	14:40	2 h 20	10:30–10:40	12:35
29 May 20	20	10:40–13:00	15:10	1 h 25	11:10–11:25	13:35
22 Jun 20	15	11:30–12:20, 12:45	15:30	1 h 15	11:15–12:05	13:55

The times to reach D_90_ in the south of the UK are at least of the order of tens of minutes. The shortest time to reach D_99_ in the south of the UK is 1 h 15, and at the end of March, it requires nearly 5 h of exposure to reach D_99_. So, the shortest times required to reach D_99_ are at least of the order of hours, rather than tens of minutes.

### Limitations and Caveats

The timescales for reaching D_90_ and D_99_ should be applicable for contaminated outdoor spaces with airborne viral load near the ground and surfaces (fomites) that are horizontal and in full view of the sky. However, consideration should be given to certain caveats. For airborne viral load, even over the shortest timescales of tens of minutes considerable dilution and dispersion would have occurred in the outdoor environment which is likely to be a significant contributing factor for reducing onward transmission (19). In addition, tens of minutes may be far too long for sufficient inactivation of virions in larger particles between emission by a cough and receipt of the airborne droplets by another person. For surface transmission, there are multiple factors that could increase the length of time required to reach this level of inactivation, including grease and dirt, replacement of viral load, inclined surfaces, surfaces facing the ground such as the underneath of a gate latch, surfaces facing north or in shade. The times for viral inactivation by solar UV shown in Fig. 5 and Table 5 should therefore be treated with caution since they are best case scenarios.

## CONCLUSION

Solar UV daily radiant exposures, in particular for the shortest UV wavelengths, were significantly higher in spring 2020 in comparison with the preceding 5 years across most of the UK, while irradiance generally appeared to be in the normal expected range of 2015–2019. The month with the greatest increases at the most locations was April, with increases in erythema effective daily radiant exposure of > 30% at all eight UK solar monitoring sites and increases in UV‐A daily radiant exposure of > 20% at five sites. The significant increase in UV daily radiant exposures in spring 2020 can be explained by a higher frequency of clear days rather than increases in irradiance.

There is evidence to suggest that the higher solar UV radiant exposures in spring 2020 increased the potential for viral inactivation outdoors in April (for reaching D_90_ in one day and 1 h and D_99_ in one day) and May (for reaching D_90_ in 1 h and D_99_ in one day) when compared to the 2015–2019 values.

Assessment of the 6‐year period 2015–2020 showed that a viral inactivation threshold of D_90_ or greater could not be reached even with a full day of exposure to solar UV for 50–60% of the year (lowest–highest UK latitudes). D_90_ in a single day was possible from April to September across the UK and not possible for most of October to March and all of November to January. D_90_ in 1 h and D_99_ in one day were possible at lower UK latitudes from April to September for up to around 75% of days. At higher UK latitudes, these levels of viral inactivation were possible from May to August for up to approximately 50% of days.

On days when D_90_ can be reached, timescales to achieve this are highly dependent on the time of year and time of day. The shortest times to reach D_90_ in the UK are of the order of tens of minutes with the latest cutoff time for reaching D_90_ before the end of the day being around 15:30 UTC. The shortest times to reach D_99_ in the UK are of the order of hours with the latest cutoff time for reaching D_99_ before the end of the day being around 14:00 UTC. However, these times assume an ideal scenario of, for example, exposure of a horizontal surface in full view of the sky for the whole day and they should therefore be treated with caution.

Overall, these findings show that for generally at least half the year in the UK solar UV is unlikely to have a significant (at least 90% inactivation) impact on viral inactivation outdoors. These results suggest that sunlight alone cannot always be relied upon to inactivate the virus outdoors in the UK.
